# Significant improvement of oxidase activity through the genetic incorporation of a redox-active unnatural amino acid†
†Electronic supplementary information (ESI) available: Experimental procedures. See DOI: 10.1039/c5sc01126d



**DOI:** 10.1039/c5sc01126d

**Published:** 2015-04-13

**Authors:** Yang Yu, Qing Zhou, Li Wang, Xiaohong Liu, Wei Zhang, Meirong Hu, Jianshu Dong, Jiasong Li, Xiaoxuan Lv, Hanlin Ouyang, Han Li, Feng Gao, Weimin Gong, Yi Lu, Jiangyun Wang

**Affiliations:** a School of Life Sciences , University of Science and Technology of China , Hefei , Anhui 230026 , China; b Laboratory of RNA Biology , Institute of Biophysics , Chinese Academy of Sciences , 15 Datun Road, Chaoyang District , Beijing , 100101 , China . Email: jwang@ibp.ac.cn; c Center of Biophysics and Computational Biology and Department of Chemistry , University of Illinois at Urbana-Champaign , Urbana , Illinois 61801 , USA . Email: yi-lu@illinois.edu

## Abstract

While nature employs various covalent and non-covalent strategies to modulate tyrosine (Y) redox potential and p*K*_a_ in order to optimize enzyme activities, such approaches have not been systematically applied for the design of functional metalloproteins. Through the genetic incorporation of 3-methoxytyrosine (OMeY) into myoglobin, we replicated important features of cytochrome c oxidase (CcO) in this small soluble protein, which exhibits selective O_2_ reduction activity while generating a small amount of reactive oxygen species (ROS). These results demonstrate that the electron donating ability of a tyrosine residue in the active site is important for CcO function. Moreover, we elucidated the structural basis for the genetic incorporation of OMeY into proteins by solving the X-ray structure of OMeY specific aminoacyl-tRNA synthetase complexed with OMeY.

## Abstract 2

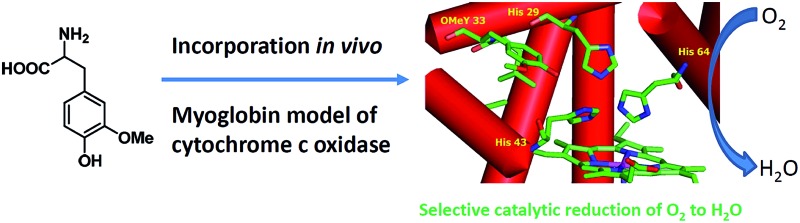
Incorporation of 3-methoxytyrosine boosts the oxidase activity of the myoglobin model of oxidase, stressing the importance of the redox potential tuning of tyrosine.

## Introduction

Designing artificial enzymes with higher activity and selectivity can reveal important features responsible for tuning enzymatic activities, and result in efficient catalysts for practical applications.^1–9^ One key mechanism which accounts for the high activity of many natural enzymes is the fine-tuning of the redox potential of tyrosine residues. In order to optimize the electron transfer rate to enable enzymatic turnover with high efficiency and selectivity, nature has exploited various strategies, such as post-translational modifications including topa quinone in copper amine oxidases, the Tyr-His crosslink in CcO, the Tyr-Cys crosslink in galactose oxidases (GO), and histidine base association in photosystem II (PSII).^10–16^ Such strategies are highly effective in modulating the redox potential (from 0.1 V to 1.1 V *vs.* NHE) and p*K*_a_ of specific tyrosine residues to suit the specific needs of various reactions, thereby greatly enhancing enzyme activity.^10–16^ However, it remains difficult to perform rational tuning of the redox potential and p*K*_a_ of specific tyrosine residues in designed metalloproteins. Here we show that, through the genetic incorporation of redox-active unnatural amino acids with desirable redox potential, a significant improvement in oxidase activity can be achieved.

During the final stage of aerobic respiration, CcO catalyzes the efficient reduction of O_2_ to H_2_O, which requires rapid transfer of four electrons and four protons to the oxygen substrate, preventing the release of toxic reactive oxygen species (ROS).^15^ The key step in oxygen reduction is the scission of the O–O bond in the ferric-superoxo intermediate, leading to the formation of an intermediate P in the heme *a*_3_/Cu_B_ binuclear active site.^17^ The donor of a proton and electron for this reaction has been suggested to be a unique tyrosine residue covalently cross-linked to one of the histidine ligands of Cu_B_. This Tyr-His crosslink is thought to lower the p*K*_a_ and redox potential of the tyrosine residue, thus facilitating proton and electron donation to the oxygen substrate.^15,18^

In previous studies, we have reported the introduction of various tyrosine analogs into a myoglobin-based functional oxidase,^19,20^ including imiTyr,^19^ which mimics the Tyr-His cross-link in CcO, and a series of halogenated Tyr analogs with decreasing p*K*_a_.^21^ By replacing Tyr33 with imiTyr and halogenated tyrosine analogs, the activity and selectivity of the functional oxidase increases. Moreover, the oxidase activity is correlated with the p*K*_a_ of the phenol ring of Tyr or its halogenated analogs, indicating the active role of Tyr in the oxidase reaction. However, since the reduction potentials of halogenated Tyr analogs are closely related to their p*K*_a_, we have not yet addressed whether fine-tuning the redox potential of the tyrosine residue influences oxidase activity. Herein we report genetic incorporation of a tyrosine analog, 3-methoxy tyrosine (OMeY), that has a lower reduction potential but similar p*K*_a_ compared to Tyr, to provide evidence that tuning the reduction potential of Tyr is also important for oxidase activity.

## Results and discussion

We first attempted to genetically incorporate two previously reported Tyr analogs, 3,4-dihydroxy-l-phenylalanine (Dopa)^22^ and 3-amino-tyrosine (NH_2_Y),^23^ which have lower redox potentials than tyrosine, into the 33rd position of Cu_B_Mb. However, yields of these mutant proteins were quite low, preventing further characterization. One explanation for these results is that Dopa and NH_2_Y can both undergo an irreversible two-electron oxidation reaction to afford dopaquinone, which is well-known to be highly reactive and to play pivotal roles in melanogenesis (Fig. S1†).^24^ This problem may be exacerbated when an oxidase or oxidase-mimicking enzyme is over-expressed in *E. coli*. In order to circumvent this problem, we decided to genetically incorporate OMeY, because its redox potential at pH 7 is 179 mV lower than that of Tyr (Fig. S2†), but it cannot easily undergo the two-electron oxidation reaction.

**Scheme 1:**
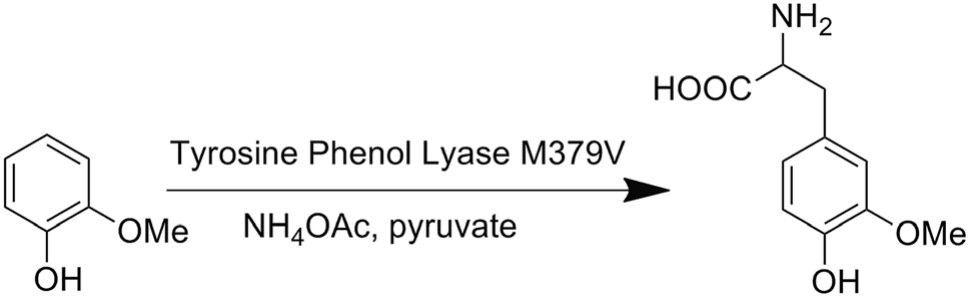
A biosynthetic route to OMeY, catalyzed by the TPL mutant M379V.

The difficulty of synthesizing unnatural amino acid analogs of Tyr, such as OMeY, is often a limiting factor for the systematic investigation of the role of Tyr in different enzymes. Enzymatic transformation by native or engineered tyrosine phenol lyase (TPL), as demonstrated herein, has been proven to be a powerful tool for synthesizing tyrosine analogs (Scheme 1).^25,26^ To synthesize OMeY, we first tried to transform 2-methoxyphenol to OMeY by using the wild type *Symbiobacterium* sp. SC-1 TPL, which is more thermostable than *Citrobacter freundii* (ATCC8090).^27^ However, we could not detect any OMeY by ninhydrin thin-layer chromatography (TLC) assay. To evolve a TPL mutant that could efficiently catalyze this transformation, we screened a TPL library pEt-*Symb*TPL, which harbors random mutations at sites Phe36, Met288, Met379, and Phe448 as previously reported,^26,28^ and found that one clone efficiently catalyzed the synthesis of OMeY, as confirmed by mass spectrometry after purification of the product by HPLC (Fig. S3–S4†). DNA sequencing revealed that this clone contains the Met379Val mutation. Molecular modeling indicated that the Met379Val mutation results in a significant enlargement of the enzyme pocket to allow for optimal interaction between the enzyme and the OMeY substrate (Fig. S5†). The reduction potential of OMeY is 179 mV lower than that of Tyr at pH 7, whereas the p*K*_a_ values of OMeY and tyrosine are similar (Fig. S2 and S6†).

To selectively incorporate OMeY at defined sites in proteins, a mutant *Methanocaldococcus jannaschii* tyrosyl amber suppressor tRNA (MjtRNACUATyr)/tyrosyl-tRNA synthetase (*Mj*TyrRS) pair was evolved that uniquely specifies OMeY in response to the TAG codon, as previously reported.^19^ The evolved TyrRS (Fig. S7–S8†), named OMeYRS, has six mutations: Tyr32Glu, Leu65Ser, His70Gly, Tyr109Gly, Asp158Asn, and Leu162Val.

**Fig. 1:**
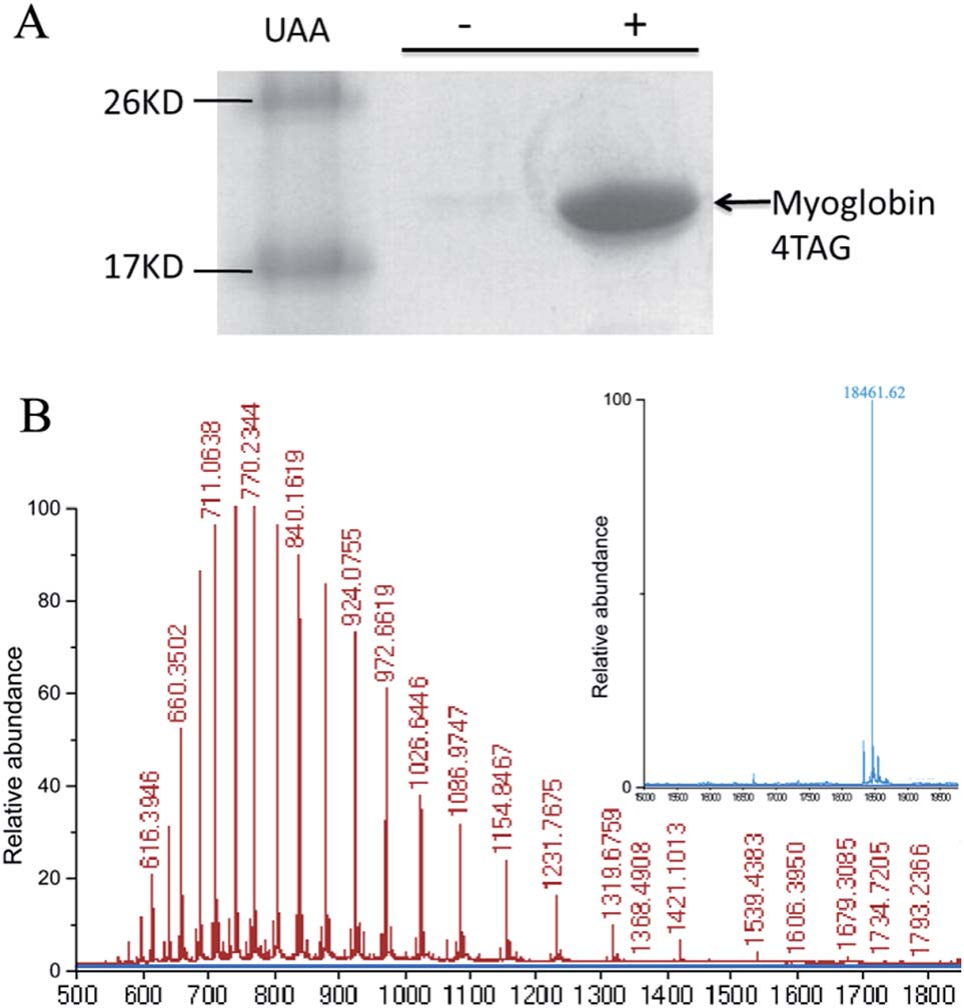
(A) SDS-PAGE of expression of TAG4 myoglobin mutant in the presence (right lane) and absence (middle lane) of 1 mM unnatural amino acid OMeY (UAA). (B) ESI-MS of the TAG4 mutant. The inset shows the deconvoluted spectrum; expected mass: 18 461 Da, found: 18 461.62 Da.

To determine if OMeY could be incorporated into proteins with high efficiency and fidelity, an amber stop codon was substituted for Ser4 in sperm whale myoglobin (Mb). Protein production was carried out in *E. coli* in the presence of the selected synthetase (OMeYRS), *Mj*tRNACUATyr and 1 mM OMeY, or in the absence of OMeY as a negative control. Analysis of the purified protein by SDS-PAGE showed that full-length myoglobin was expressed only in the presence of OMeY (Fig. 1A), indicating that OMeYRS was specifically active with OMeY but inactive with natural amino acids. The yield for this mutant myoglobin was 10 mg L^–1^. By comparison, the yield of wild-type sperm whale myoglobin (WTMb) was 50 mg L^–1^. ESI-MS analysis of the Ser4 OMeY mutant myoglobin gave an observed average mass of 18 461.6 Da, in agreement with the calculated mass of 18 461.1 Da (Fig. 1B).

**Fig. 2:**
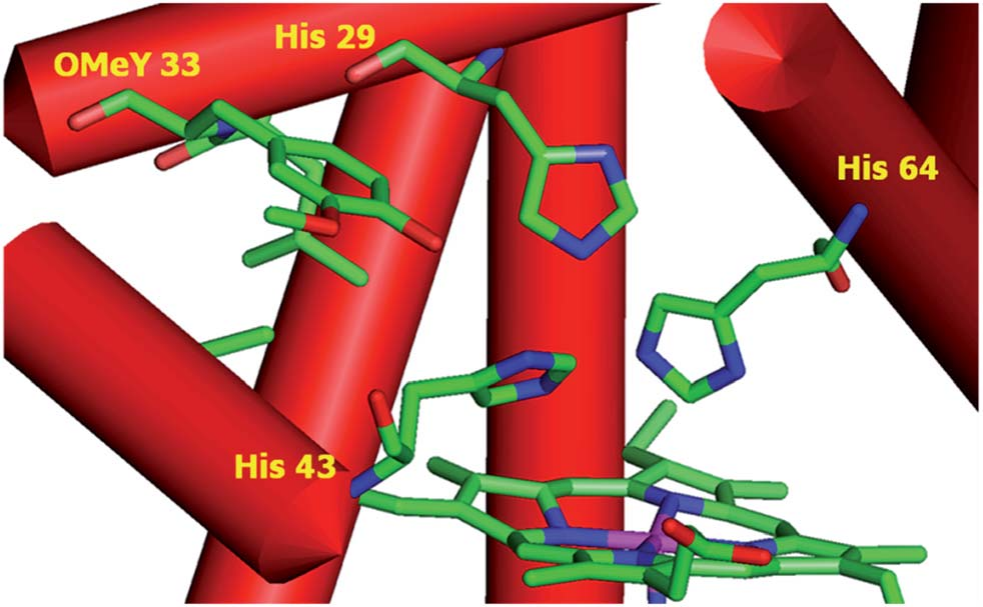
Structural model of OMeY myoglobin mutant, constructed based on the crystal structure of Phe33Tyr-Cu_B_Mb (pdb code ; 4FWX).

To test whether the catalytic activity could be improved through the genetic incorporation of unnatural amino acids, we replaced Phe33 in Cu_B_Mb with OMeY, generating Phe33OMeY-Cu_B_Mb (Fig. 2). This mutant showed a similar UV-vis spectrum to that of Phe33Tyr-Cu_B_Mb (Fig. S9†), indicating that the overall environment around the heme center should also be similar.^20^

**Fig. 3:**
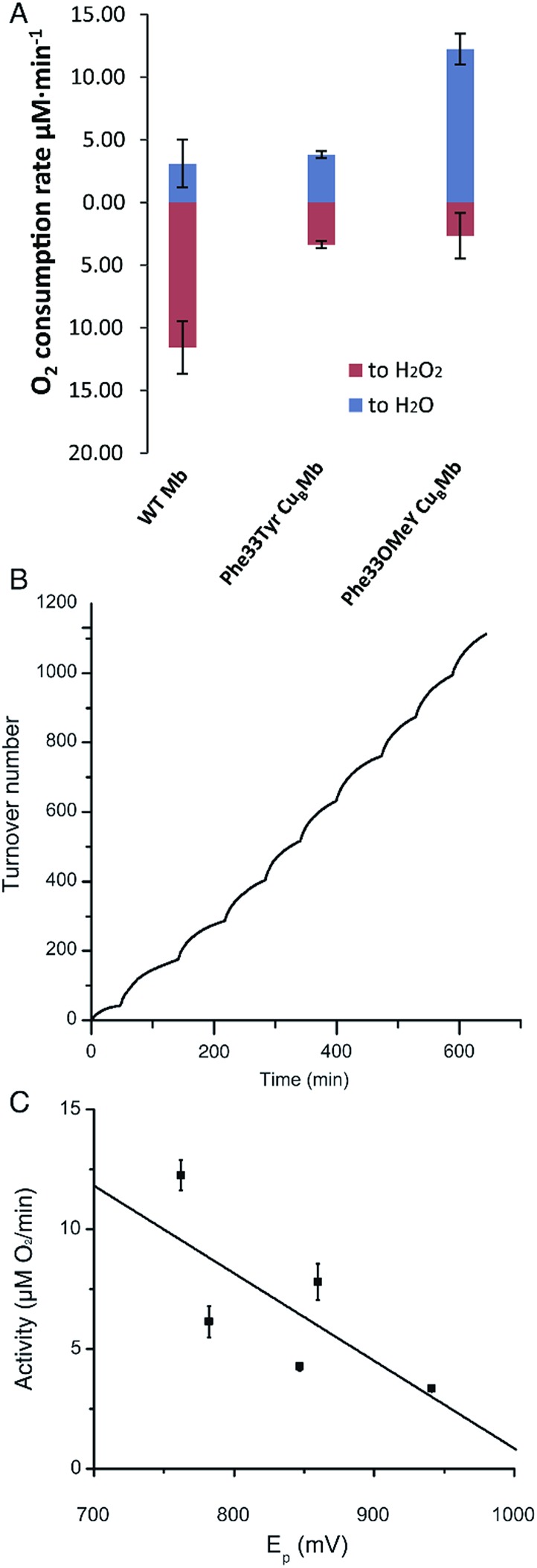
(A) Rates of oxygen reduction to form either water (blue) or ROS (red) catalyzed by 6 μM WTMb, Phe33Tyr-CuBMb or Phe33OMeY-Cu_B_Mb. (B) O_2_ reduction turnover number for Phe33OMeYCu_B_Mb, measured during the stepwise addition of O_2_. (C) Plot of oxidase activity of Phe33Tyr-Cu_B_Mb, Phe33ClY-Cu_B_Mb, Phe33F_2_Y-Cu_B_Mb, Phe33F_3_Y-Cu_B_Mb, and Phe33OMeY-Cu_B_Mb *vs.* the peak potential at pH 7 (*E*_p_) of the corresponding Tyr and Tyr analogs. *E*_p_ values measured by cyclic voltammetry were 941 mV for Tyr, 847 mV for ClY, 782 mV for F_2_Tyr, 860 mV for F_3_Y, and 762 mV for OMeY. Abbreviations: ClY, 3-chlorotyrosine; F_2_Y, 3,5-difluorotyrosine; F_3_Y, 2,3,5-trifluorotyrosine.

We then measured the rates of oxygen reduction catalyzed by 6 μM myoglobin mutants with an O_2_ electrode in 20 mM tris(hydroxymethyl)aminomethane (Tris) buffer at pH 7.4. Ascorbate (1000 equivalents) and tetramethyl-*p*-phenylenediamine dihydrochloride (TMPD, 100 equivalents) were used as reductant and redox mediator, respectively.^29^ To differentiate between reactive oxygen species (ROS) and water products, we used catalase and superoxide dismutase (SOD), which catalyze the disproportionation of hydrogen peroxide or superoxide into oxygen and water. If O_2_ consumption results in the formation of ROS but not water, the O_2_ reduction rate should decrease in the presence of catalase and SOD, because they will convert ROS to O_2_. By comparing the rates of reduction in the presence and absence of ROS scavenger, the portions of O_2_ reduction due to water formation (in blue) and due to ROS formation (in red) can be calculated (Fig. 3A and Table S2†). Our results show that Phe33Tyr-Cu_B_Mb was able to reduce O_2_ at a rate of 6.5 μM min^–1^, with 51% of O_2_ being converted into water. In contrast, Phe33OMeY-Cu_B_Mb exhibited significantly higher oxidase activity at 15.0 μM min^–1^ for O_2_ reduction, with 82% conversion of O_2_ into water. Similarly to the case of Phe33Tyr-Cu_B_Mb, addition of copper to Phe33OMeY-Cu_B_Mb did not increase oxidase activity.^20^ Since the p*K*_a_ of OMeY (Fig. S6†) is similar to that of Tyr, the lower redox potential of OMeY is likely responsible for the increased oxidase activity and O_2_ reduction selectivity of Phe33OMeY-Cu_B_Mb.

To further demonstrate the robustness of the best oxidase-mimicking enzyme, Phe33OMeY-Cu_B_Mb, we carried out multiple turnover experiments (Fig. 3B). Phe33OMeY-Cu_B_Mb was able to catalyze O_2_ reduction for more than 1100 turnovers without significant reduction of catalytic rate. Under similar conditions, Phe33Tyr-Cu_B_Mb could only catalyze the reaction for fewer than 500 turnovers.^20^

Previous studies with various halogenated Tyr analogs in an oxidase model have shown that oxidase activity is correlated with the p*K*_a_ of Tyr or its analogs, however, correlation between oxidase activity and reduction potential at pH 7 is weak. Since Tyr oxidation at neutral pH, when Tyr is protonated, is a process coupled with the loss of a proton, the reduction potential of Tyr is influenced by p*K*_a_. It is hard to separate the effect of p*K*_a_ from the reduction potential of Tyr. As OMeY has a much lower reduction potential but similar p*K*_a_ to Tyr, it is clear that decreasing the reduction potential, similar to decreasing the p*K*_a_, also enhances oxidase activity (Fig. 3C). The correlation of reduction potential/p*K*_a_ with oxidase activity is consistent with the active role of Tyr in the oxidase reaction, as previous studies have shown that a tyrosyl radical is formed during the oxygen reduction reaction of the Mb-based functional oxidase.^30^

Unnatural Tyr analogs as spectroscopic or functional probes have been developed to study the function of Tyr in different enzymes. Halogenated Tyr analogs have different p*K*_a_ values, as well as distinct EPR signals,^21,25^ making them useful for pin-pointing the location of the tyrosyl radical intermediate and the proton donating ability of Tyr. Dopa and NH_2_Y have decreased reduction potential.^22,23^ They are used in reductive enzymes as they are susceptible to oxidative damage. OMeY has a lower reduction potential than Tyr, yet is relatively stable under oxidative conditions, making it suitable for studying oxidative enzymes, such as cytochrome c oxidase, galactose oxidase, and lytic polysaccharide monooxygenase.

## Conclusions

In summary, by incorporation of OMeY, an analog with a 179 mV lower reduction potential and similar p*K*_a_ to Tyr, into a Mb-based functional oxidase, we found that the oxidase activity of the protein is correlated with the reduction potential of active site Tyr or its analogs. This further reveals the active role of Tyr in the oxidase reaction.

Tyr is an important residue for electron transfer^31^ as well as catalysis,^14^ due to its redox activity and proton-coupled electron transfer ability. Nature has evolved different ways of conducting post-translational modifications,^32–34^ along with manipulating hydrogen bonding and π–π stacking to fine-tune the properties of Tyr. OMeY, with its low reduction potential while being relatively stable to O_2_, has been added as a unique member to the toolbox of Tyr analogs^35–37^ for studying and engineering Tyr-containing enzymes.
