# Supplementary material for: Significant improvement of oxidase activity through the genetic incorporation of a redox-active unnatural amino acid
Source: Chem Sci. 2015 Apr 13;6(7):3881–5. doi: 10.1039/c5sc01126d (PMC4583198; doi:10.1039/c5sc01126d)
Supplement: Supplementary file 1 [file SC-006-C5SC01126D-s001.pdf]

Electronic Supplementary Material (ESI) for Chemical Science.
This journal is © The Royal Society of Chemistry 2015

Supporting information for

Significant Improvement of Oxidase Activity through the Genetic Incorporation of Redox-active Unnatural Amino Acids

Yang Yu[c‡], Qing Zhou[b‡], Li Wang[a,b‡], Xiaohong Liu[b], Wei Zhang[b], Meirong Hu[b], Jianshu Dong[b], Jiasong Li[a,b], Xiaoxuan Lv[b], Hanlin Ouyang[c], Han Li[b], Feng Gao[b], Weimin Gong[b], Yi Lu*[c], Jiangyun Wang*[b]

[a] School of Life Sciences, University of Science and Technology of China, Hefei, Anhui, 230026, China.

[b] Laboratory of Non-coding RNA, Institute of Biophysics, Chinese Academy of Sciences, 15 Datun Road, Chaoyang District, Beijing, 100101, China

[c] Center of Biophysics and Computational Biology and Department of Chemistry, University of Illinois at Urbana-Champaign, Urbana, Illinois, 61801, USA

Corresponding Authors
E-mail: jwang@ibp.ac.cn, yi-lu@illinois.edu

Author Contributions
‡These authors contributed equally to this work.

Materials.

2-methoxyphenol was purchased from Jkchemical. All other chemicals were purchased from Sigma-Aldrich and used without further purification. ^{1}H NMR spectra were recorded on a Bruker AMX-400 instrument with chemical shifts reported relative to tetramethylsilane. Protein mass spectra were run on a thermo LTQ-orbitrap at the IBP, CAS (Beijing, China) or Waters Quattro II electrospray quadrupole-hexapole-quadrupole (QHQ) mass spectrometer at mass

spectrometry lab of University of Illinois at Urbana-Champaign. Anaerobic buffers were prepared by boiling for 5 min followed by argon purging until cooled to room temperature.

Synthesis of 2-amino-3-(4-hydroxy-3-methoxyphenyl)propanoic acid 1: A 1 liter solution containing 30 mM ammonium acetate, 10 mM 2-methoxyphenol, 60 mM sodium pyruvate, 5 mM b- mercaptoethanol, 40 mM PLP, 10 milligram TPL Phe36His mutant enzyme, pH 8.0, was stirred in the dark for 3 days at room temperature. The aqueous phase was then concentrated, and was purified by HPLC to afford a white powder at 40% yield (YMC AA12S052503WT column, 12 ml/min flow rate, from 10% to 90% CH3CN, 0.1% TFA (w/v) in water, over the course of 30 min). MS: m/z: 212 [M + H]+; H-NMR (400 MHz, DMSO-d6): 3.74 (s, 3H), 3.16 (m, 2H), 4.18 (t, 1H), 6.68 (d, 1H, J = 8.1), 6.78 (dd, J = 8.1, 1.96, 1H), 6.83 (d, J = 1.96, 1H)

pK_a measurement.

pK_a values of phenol and substituted phenols were determined spectrophotometrically by monitoring the bathochromic shift (ca. 270 nm to 290 nm) that occurs upon conversion of the neutral phenol to the phenolate anion, as described before.[1] The data was fit into Hill equation to get cooperativity close to 1 and pK_a was calculated.

$$\theta = \frac{(10^{-pH})^n}{10^{-pK_a} + (10^{-pH})^n}$$

Redox potential measurement.

Oxidation of tyrosine and tyrosine analogues were measured by cyclic voltammetry as described before.[2] The measurement was performed on a CH Instruments 617A potentiostat equipped with a picoamp booster and a Faraday cage. Amino acids were dissolved in UB buffer to make 200μM solution. pH of solution was adjusted before put into system. The solution was

put into 3-electrode chamber with a glass carbon working electrode, a Ag/AgCl reference electrode and a Pt auxiliary electrode. The system was purged with Ar for 2 min before measurement. CV parameters were as follows: scan rate: 10mV/s, sample interval: 1mV, sensitivity: 10μA/V, quiet time: 4s, temperature: 25 °C.

Plasmids and cell lines used.

Plasmid pBK-lib-jw1 encodes a library of *M. Jannaschii* tyrosyl tRNA synthetase (TyrRS) mutants randomized at residues Tyr32, Leu65, Phe108, Gln109, Asp158 and Leu162; and any one of the six residues (Ile63, Ala67, His70, Tyr114, Ile159, Val164) was either mutated to Gly or kept unchanged. Plasmid pREP(2)/YC encodes *Mj*tRNA$_{CUA}^{Tyr}$, the chloramphenicol acetyltransferase (CAT) gene with a TAG codon at residue 112, the GFP gene under control of the T7 promoter, and a Tetr marker; plasmid pLWJ17B3 encodes *Mj*tRNA$_{CUA}^{Tyr}$ under the control of the *lpp* promoter and *rrnC* terminator, the barnase gene (with three amber codons at residues 2, 44 and 65) under the control of the *ara* promoter, and an Amp^{r} marker. Plasmid pBAD/JYAMB-4TAG encodes the mutant sperm whale myoglobin gene with an arabinose promoter and *rrnB* terminator, *Mj*tRNA$_{tyr}^{CUA}$ with an *lpp* promoter and *rrnC* terminator, and a tetracycline resistance marker; E. coli strain GeneHog®-Fis, F- *mcr*A (*mrr-hsd*RMS-*mcr*BC) 80*lac*Z M15 *lac*X74 *rec*A1 *end*A1 *ara*D139 (*ara-leu*)7697 *gal*U *gal*K *rps*L(StrR) *nup*G, *fis*::Tn7 (DHFR).

Ninhydrin test.

To test for the presence of the unnatural amino acid, a ninhydrin solution consisting of 0.2% (w/v) ninhydrin, 95% (v/v) n-butanol, 0.5% (v/v) acetic acid and 4.3% (v/v) water was

prepared. The enzymatic reaction mixture was first mixed with the ninhydrin solution at a 1:1 ratio, and then spotted onto a TLC plate. A purple/pink spot indicates the presence of the unnatural amino acid.

Genetic selection of the mutant synthetase specific for OMeY 1.

pBK-lib-jw1 consisting of 2×10^9 TyrRS independent clones was constructed using standard PCR methods. E. coli DH10B harboring the pREP(2)/YC plasmid was used as the host strain for the positive selection. Cells were transformed with the pBK-lib-jw1 library, recovered in SOC for 1 h, washed twice with glycerol minimal media with leucine (GMML) before plating on GMML-agar plates supplemented with kanamycin, chloramphenicol, tetracycline and 1 at 50 mg/mL, 60 mg/mL, 15 mg/mL and 1 mM respectively. Plates were incubated at 37 °C for 60 hours, surviving cells were scraped, and plasmid DNA was extracted and purified by gel electrophoresis. The pBKlib-jw1 DNA was then transformed into electro-competent cells harboring the negative selection plasmid pLWJ17B3, recovered for 1 h in SOC and then plated on LB-agar plates containing 0.2% arabinose, 50 mg/mL ampicillin and 50 mg/mL kanamycin. The plates were then incubated at 37 °C for 8-12 h, and pBK-lib-jw1 DNA from the surviving clones was extracted as described above. The library was then carried through a subsequent round of positive selection, followed by a negative selection and a final round of positive selection (with chloramphenicol at 70 mg/mL). At this stage, 96 individual clones were selected and suspended in 50 mL of GMML in a 96-well plate, and replica-spotted on two sets of GMML plates. One set of GMML-agar plates was supplemented with tetracycline (15 mg/mL), kanamycin (50 mg/mL) and chloramphenicol at concentrations of 60, 80, 100 and 120 mg/mL

with 1 mM 1. The other set of plates were identical but did not contain **1**, and the chloramphenicol concentrations used were 0, 20, 40 and 60 mg/mL. After 60 h incubation at 37 °C, one clone was found to survive at 100 mg/mL chloramphenicol in the presence of 1 mM **1**, but only at 20 mg/mL chloramphenicol in the absence **1**.

Mutant myoglobin expression.

To express mutant myoglobin protein, plasmid pBADJYAMB-Leu29HisPhe33TAGPhe43His was cotransformed with pBK-ClTyrRS/ MoxTyrRS into TOP10 *E. coli* competent cells. Cells were amplified in LB media (5 mL) supplemented with kanamycin (50 μg/mL) and tetracycline (15 μg/mL). A starter culture (1 mL) was used to inoculate 100 mL of liquid LB supplemented with appropriate antibiotics and corresponding unnatural amino acid (1 mM). Cells were then grown at 37°C to OD600 (optical density at 600 nm) of 0.5, and protein expression was induced by the addition of 0.2% arabinose. After 12 h of growth at 37°C, cells were harvested by centrifugation. Wild-type or mutant myoglobin was then purified by Ni-NTA affinity chromatography and size exclusion chromatography to achieve homogeneity.

Oxygen consumption assay.

Oxygen consumption was measured by using an Oxygraph Clark-type oxygen electrode (Hansatech Instruments) at 25°C in 20 mM Tris buffer, pH 7.4 similar to described before.[3,4] All experiments were performed three times to obtain standard deviation. The electrode was calibrated against air-saturated buffer and O_2-depleted buffer prior to use. Protein was exchanged to 20 mM Tris, pH7.4. The buffer was treated with chelex beads overnight before use. The final concentration of protein was adjusted to 6 μM. For experiment with Cu^{2+}, $CuSO_4$ was added to final concentration of 6 μM and stirred for 10 min. For experiment with catalase,

catalase (Sigma-Aldrich) was added to final concentration of 7.3 U/μL. The reaction is initiated by adding TMPD and ascorbic acid to a final concentration of 0.6 mM and 6 mM, respectively. Oxygen concentration was read immediately after adding reductant.

OMe-TyrRS expression, purification and homogeneity analysis

OMe-TyrRS gene from *Methanococcus jannaschii* was cloned into pET22b plasmid with the restriction endonuclease cleavage sites of NdeI and XhoI. Compared to wild type TyrRS, OMe-tyrRS (3-o-methyl tyrosyl tRNA synthetase) has the following mutation sites: Y32E, L65S, H70G, Q109G, D158N, and L162V. Ome-TyrRS-pET22b vector was transformed into BL21(DE3) host cell. OMe-tyrRS then was over expressed with c terminal his×6 tag after 3 hours of induction by 1 mM IPTG at 37ºC when OD600 was about 0.8. The harvested cell pellet was resuspended in 50 mM HEPES pH 7.9, 0.5 M NaCl, 10% glycerol, 5 mM β-mercaptoethanol，1 mM PMSF and stored at -30ºC. Cells were disrupted by sonication on ice in 50mM HEPES PH7.9, 0.5M NaCl 10% glycerol, 5 mM β-mercaptoethanol and 1 mM PMSF. After centrifugation at 4ºC for 50 minutes, target protein in the supernatant was captured by Ni-IDA affinity gel and eluted by gradient imidazole solution. Protein sample was concentrated and applied to gel filtration using superdex200 (24ml) column, the peak containing the target protein was at approximately 14ml, which indicates OMe-TyrRS dimerization. The purified protein was concentrated and stored at -80ºC before crystallization experiment. OMe-TyrRS protein sample's homogeneity was analyzed both by Native PAGE and dynamic light scattering in the 25 mM Tris-Cl pH8.0, 50 mM NaCl buffer.

OMe-TyrRS crystallization, data collection and structure determination

OMe-TyrRS crystals were obtained at 16 ºC by sitting-drop vapor diffusion method by mixing 1 μL protein sample (40 mg/ml) in 25 mM Tris-Cl pH8.0 50 mM NaCl with equal volume of reservoir solution containing 20-22% PEG3350 0.2 M $(NH_4)_2SO_4$ 0.1 M NaCitrate pH6.1-6.2. Crystals up to 0.6 mm×0.3 mm×0.2 mm in dimension were obtained within 5 days. No extra cryo-protectant was added before crystal fishing and flash-frozen in liquid nitrogen. The 3'o-methyl-Tyrosine containing structure of OMe-TyrRS was obtained through co-crystallization by sitting drop vapor diffusion technique under the same condition as apo protein. The o-methyl-Tyrosine's final concentration in the drop is about 60 mM. Diffraction data was collected at the wavelength of 0.979 Å at BL17U of the Shanghai Synchrotron Radiation Facility using marCCD detector. Data processing and reduction were carried out using the HKL2000 package. The initial model of OMe-TyrRS was solved by molecular replacement with Molrep using the atomic coordinates of apo-TyrRS from *Methanococcus jannaschii* (PDB code: 1U7D) as search model. Molecular-replacement solutions were modified by manual building into composite electron density map using Coot, and improved by iterative cycles of refinement using REFMAC, CNS and phenix.refine. The final models of apo OMe-TyrRS and 3'o-methyl-Tyrosine bound OMe-TyrRS complex were checked for geometrical correctness with PROCHECK. Since the poor electron densities for the side chains of residues E259, K260, and F261 of OMe-TyrRS complex structure, and residues E18, R139, V164, K209, E259, and K260 of apo TyrRS, these residues were mutated to Alanine in the final model. Since the poor electron densities of residues M1, E140, D141, and E142 of apo TyrRS, they were omitted in the final model. Data collection and structure refinement statistics are summarized in Table S3. Cartoon and other protein structure representations were generated using PyMOL(http://www.pymol.org).

The atomic coordinates and the structure factors have been deposited in the Protein Data Bank (PDB code: 4HK4, 4HPW).

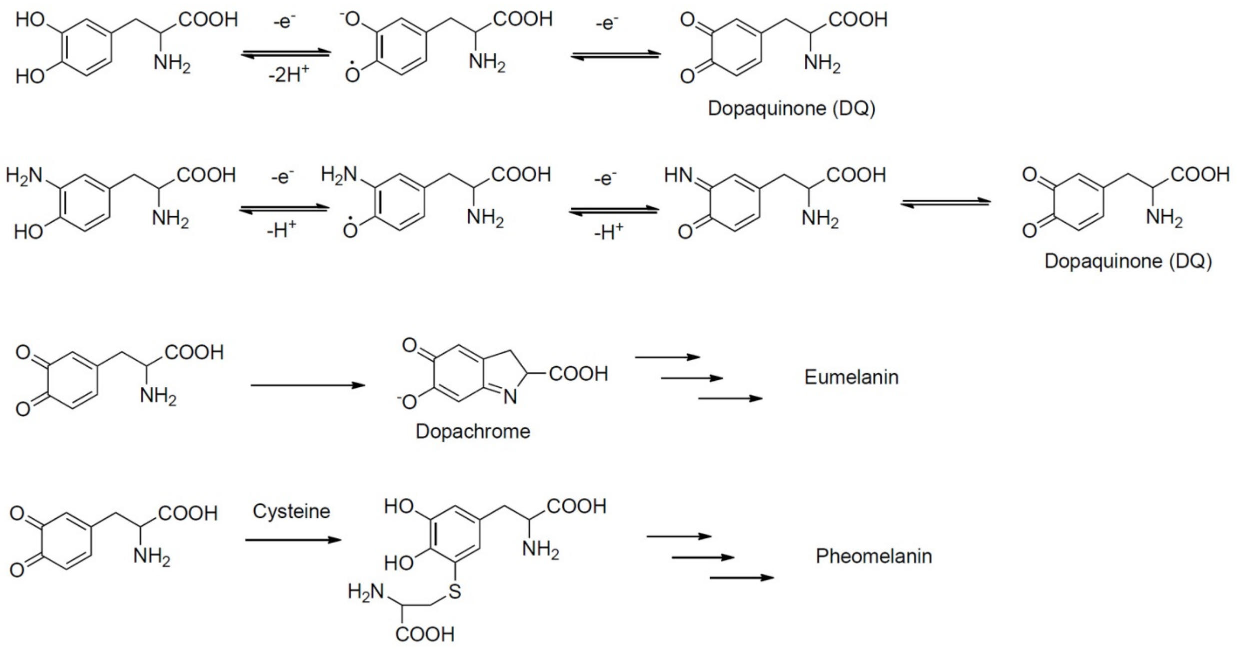

Fig. S1. Mechanism of Dopa and NH_2Y oxidation.

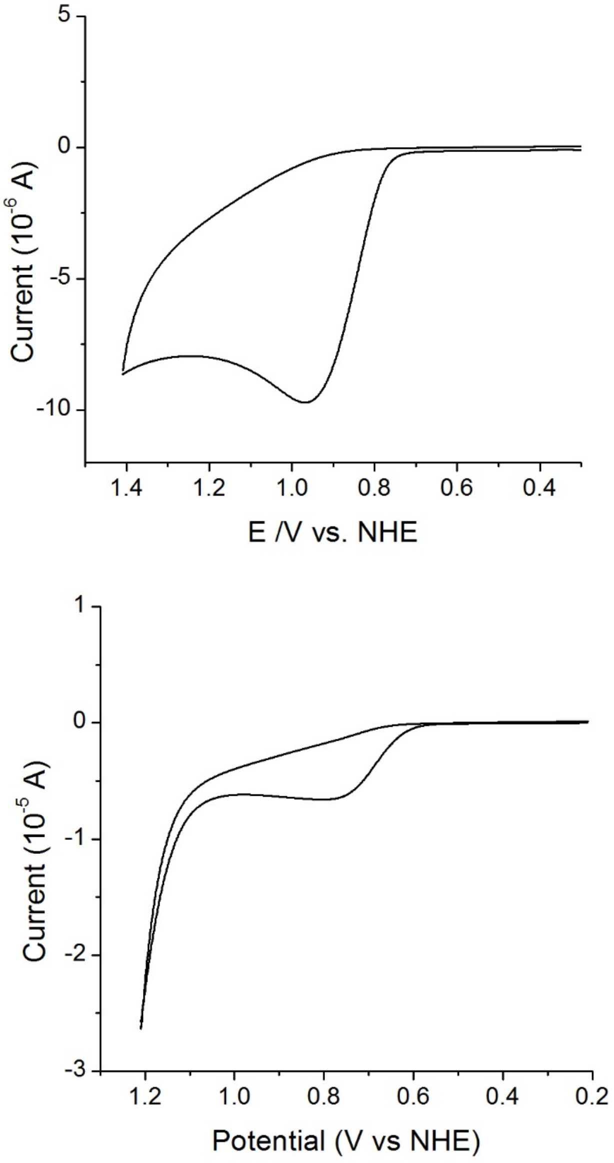

Fig. S2. Cyclic voltammetry of Tyr(top) and OMeY(bottom) at pH7.

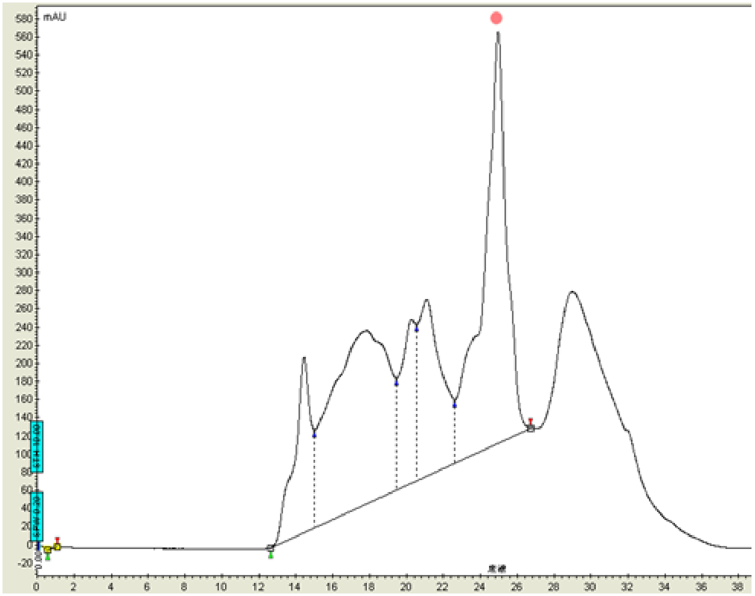

Fig. S3. HPLC purification of OMeY.

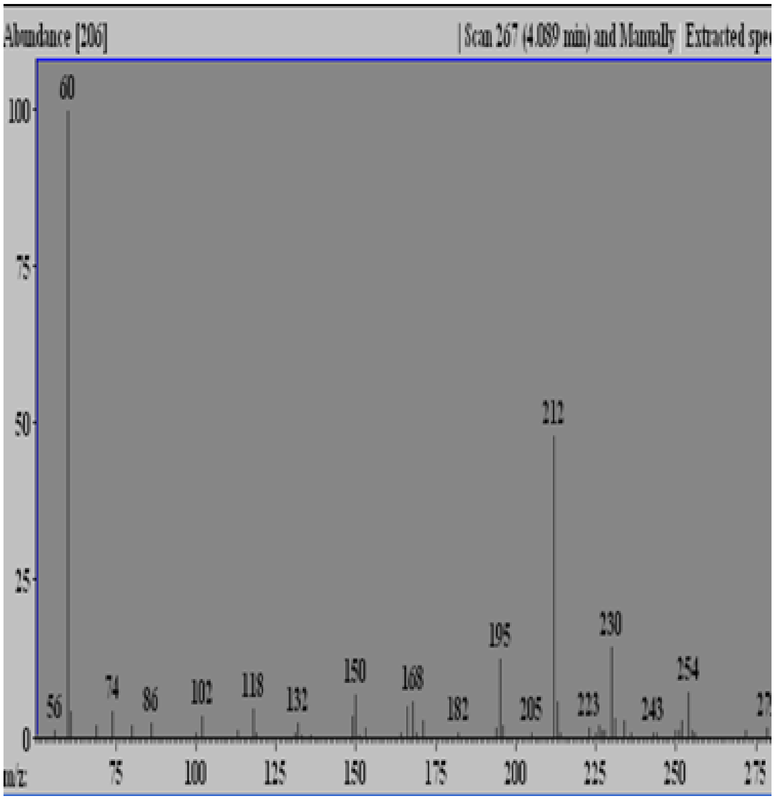

Fig. S4. ESI-MS spectrum of OMeY.

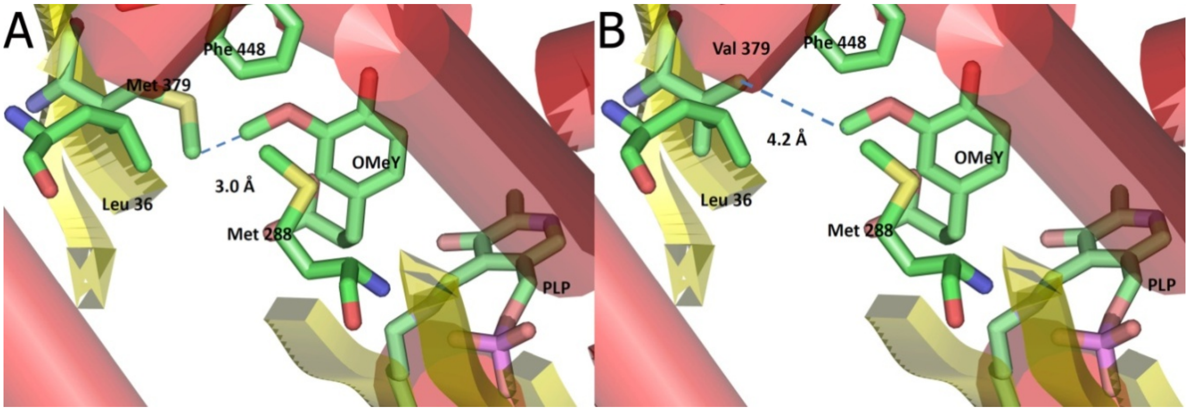

Fig. S5. Structural model of wild type TPL (A) and M379V mutant TPL (B) in complex with OMeY.

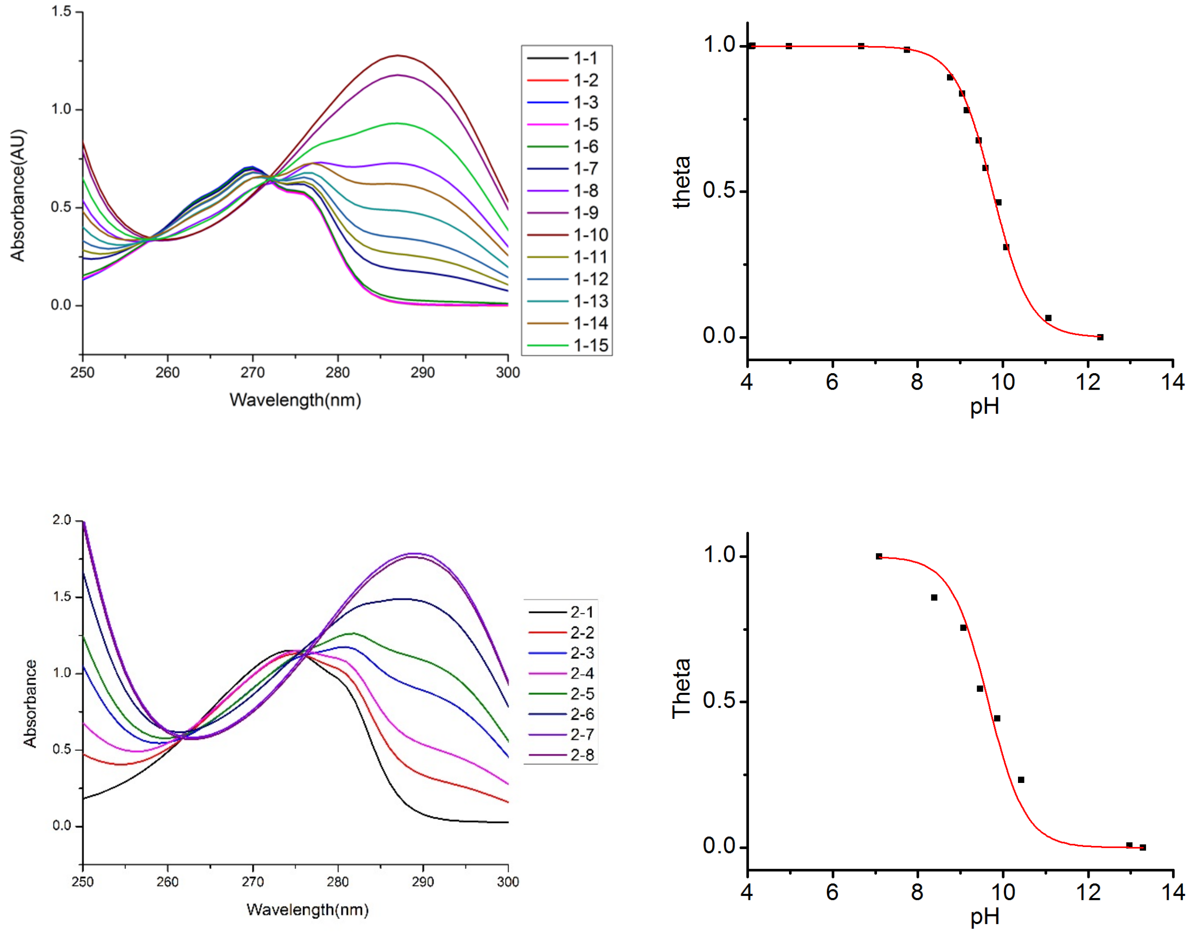

Fig. S6. pK_a measurement of phenol (top, pK_a = 9.77±0.01) and o-methoxyphenol (bottom, pK_a = 9.65±0.06). Left panel: UV absorption spectra at different pH; right panel: plot of pH and fraction of deprotonated species (filled square) and fitting to Hill equation (red line).

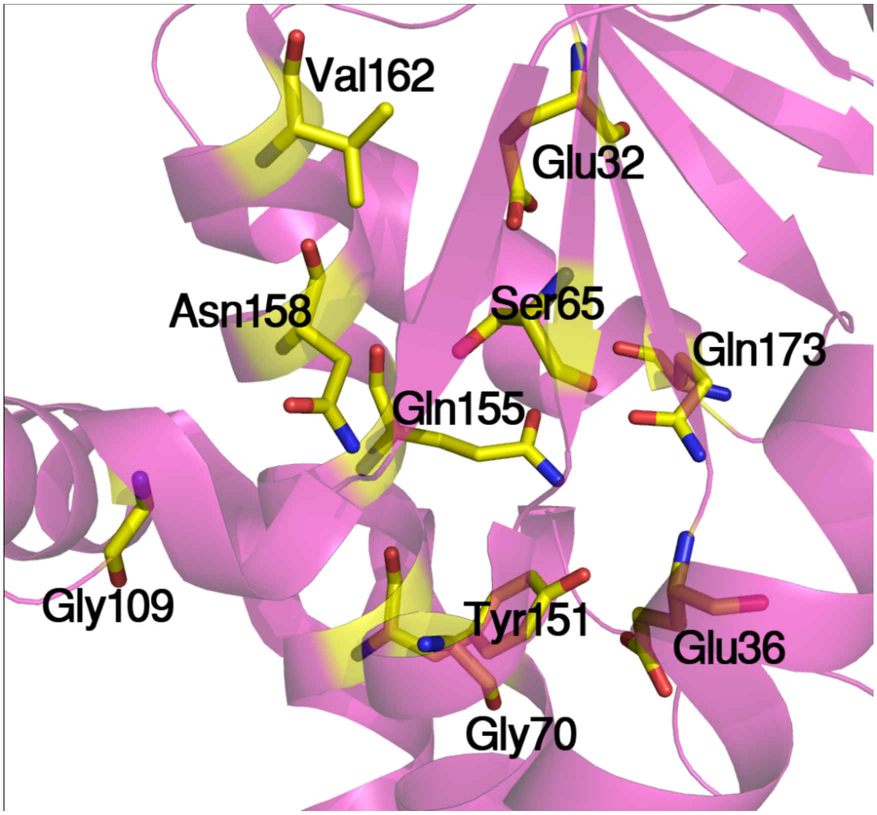

Fig. S7. TyrRS apo structure showing the active site and mutation sites

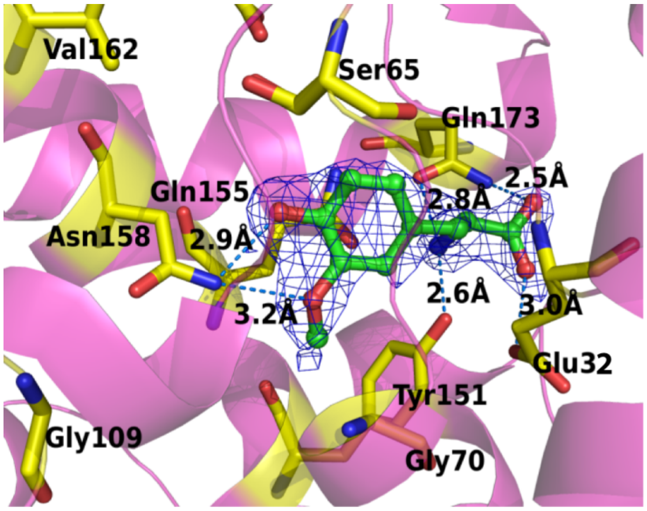

Fig. S8. Structure of OMeYRS with OMeY bound in the active site. The Fo–Fc electron density map of OMeY was contoured at 1.0σ. With Tyr32, Leu65, His70, Tyr109 and Leu162 each mutated to smaller residues, an extra space in the active site was created to accommodate the larger OMeY substrate. The mutation of Asp158 to Asn allowed the formation of hydrogen bonds with both phenol and methoxy groups of OMeY, thus increasing the affinity for OMeY.

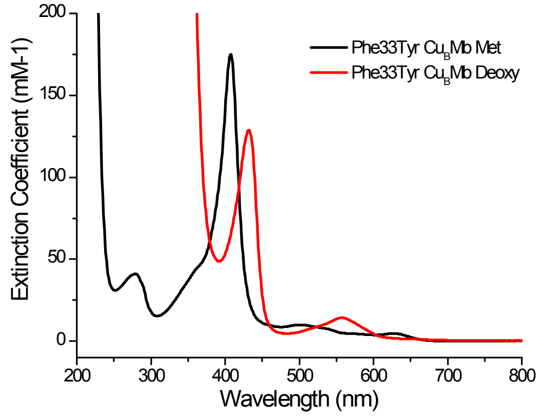

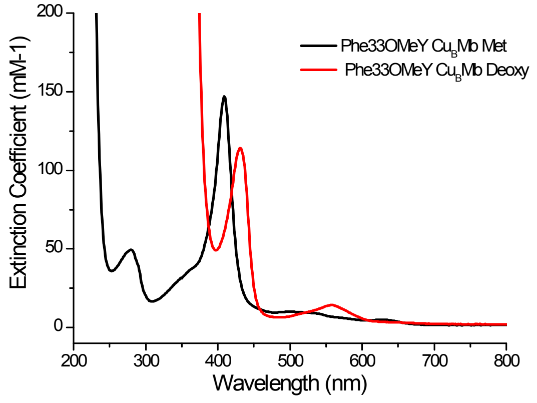

Fig. S9. UV-vis spectra of ferric (black trace) and deoxy (red trace) form of Phe33Tyr Cu_BMb(top) and Phe33OMeY Cu_BMb(bottom) respectively.

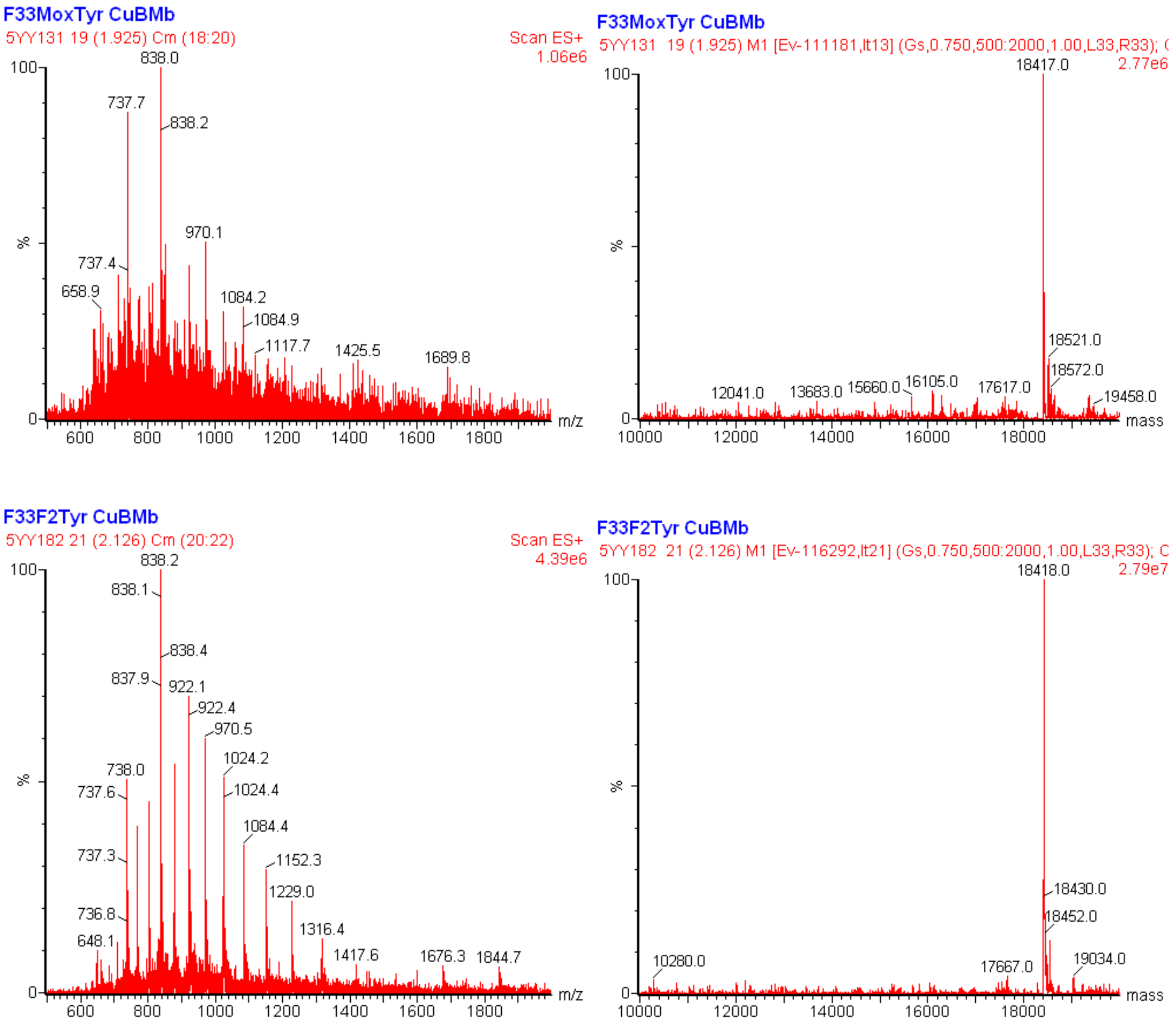

Fig. S10. Mass spectra of Phe33OMeY Cu_BMb(top) and Phe33F_2Y Cu_BMb(bottom). Phe33OMeY Cu_BMb, calculated: 18416, observed 18417; Phe33F_2Y Cu_BMb calculated:18421, observed:18418 .

Table S1. Features of myoglobins on UV-vis spectra.

	Met			Deoxy			
	Soret	Extinc. Co. mM-1	Vis feature	Soret	Extinc. Co. mM-1	Deoxy	Extinc. Co. mM-1

Phe33Tyr Cu_BMb	408	175	501, 533,628	433	128	559	14.2
Phe33F_2Y Cu_BMb	409	147	500, 537, 630	431	114	559	14.3
Phe33OMeY Cu_BMb	407	177	501, 536, 628	431	127	556	15.1

Table S2 Oxygen consumption calculation

	Phe33Tyr-Cu_BMb	Phe33Tyr-Cu_BMb+cata	Phe33OMeY-Cu_BMb	Phe33OMeY-Cu_BMb+cata
Average oxygen consumption rate (μM/min)	6.54[a]	4.95[b]	15.06[c]	13.41[d]
Standard deviation (SD)	0.05	0.14	0.70	0.52
O_2 consumption to H_2O[e]	3.36		11.77	
O_2 consumption to H_2O_2[f]	3.18		3.29	
SD_H_2O	0.28		1.25	
SD_H_2O_2	0.29		1.75	

[a] sample size n=3

[b] sample size n=3

[c] sample size n=4

[d] sample size n=4

[e] Calculation of rate of O_2 consumption towards H_2O production: 2*k(with catalase)-k(without catalase). Further addition of SOD doesn't change rate of O_2 consumption so we conclude that superoxide is not generated in the system, as shown before[6].

[f] Calculation of rate of O_2 consumption towards H_2O_2 production: 2*[k(without catalase)-k(with catalase)]

[g] Standard deviation were calculated based on propagation of uncertainty. Given rates of O_2 consumption towards H_2O or H_2O_2 production are linear combination of rate of O_2 consumption with or without catalase, $k = \sum_i^n A_i k_i$, with the assumption that the errors on k_i are not correlated,

$$\delta_k = \sqrt{\sum_i^n A_i^2 \delta_{k_i}^2}$$

Table S3. Statistics of data collection and structure determination of TyrRS-OMeY complex and apo-TyrRS

**Data collection**		
Data sets	TyrRS-OMeY	Apo-TyrRS
Space group	P2	
Unit cell parameters	a = 52.6 Å,<br>b = 38.8 Å,<br>c = 82.9Å<br>α = γ =90°<br>β =90.8°	a = 52.7 Å,<br>b = 38.9 Å,<br>c = 83.1Å<br>α = γ =90°<br>β =91.0°
Resolution range (Å)	30-2.00(2.07-2.00)	50-2.3(2.38-2.30)
No. of total reflections	159216 (15752)	72600(4558)
Average Redundancy	7.0(6.9)	5.0(4.3)
I/ σ	13.3(8.2)	30.4(5.0)
Completeness (%)	98.1(100)	94.8(70.5)
Rmerge(%)[a]	8.7(20.0)	5.3(26.5)
**Structure refinement**		
Resolution (Å)	26.3-2.0	31.3-2.3
Rcryst/ Rfree(%)[b]	20.2/24.2	18.3/24.9
**r.m.s.deviations**		
bond lengths (Å)	0.002	0.007

bond angles (°)	0.5	1.0
**B-factors**		
Protein	35.2	48.3
Ligand	62.0	--
Water	35.4	44.3
**Ramachandran plot**		
most favored regions (%)	97.1	97.7
additionally allowed (%)	2.3	2.0
generously allowed (%)	0.6	0.3

[a] $R_{merge} = \sum|I_i - I_m|/\sum I_i$, where I_i is the intensity of the measured reflection and I_m is the mean intensity of all symmetry related reflections.

[b] $R_{cryst} = \Sigma||F_{obs}| - |F_{calc}||/\Sigma|F_{obs}|$, where F_{obs} and F_{calc} are observed and calculated structure factors.

$R_{free} = \Sigma_T||F_{obs}| - |F_{calc}||/\Sigma_T|F_{obs}|$, where T is a test data set of about 5% of the total reflections randomly chosen and set aside prior to refinement.

Numbers in parentheses represent the value for the highest resolution shell.

Reference

(1) Kim, K.; Cole, P. A. *J. Am. Chem. Soc.* **1998**, *120*, 6851.

(2) Harriman, A. *J. Phys. Chem.* **1987**, *91*, 6102.

(3) Liu, X.; Yu, Y.; Hu, C.; Zhang, W.; Lu, Y.; Wang, J. *Angew. Chem. Int. Ed.* **2012**, *51*, 4312.

(4) Miner, K. D.; Mukherjee, A.; Gao, Y.-G.; Null, E. L.; Petrik, I. D.; Zhao, X.; Yeung, N.; Robinson, H.; Lu, Y. *Angew. Chem. Int. Ed.* **2012**, *51*, 5589.
